# Medical Items

**Published:** 1913-07

**Authors:** 


### MEDICAL ITEMS

The Budwell Pharmacal Co., of Lynchburg, Va., claim two particularly distinctive features for their preparation, Budwell's Emulsion of Cod Liver Oil. The most important is palatability, the emulsion being so manipulated and flavored as to make it not only easily assimilated but capable of being retained by the most delicate stomachs. The second distinctive feature is that Budwell's Emulsion is strictly an ethical preparation, making appeal only to the medical profession for whose use and convenience the emulsion is prepared. The endorsement of the medical profession has created a demand for Budwell's Emulsion in practically every state of the United States. The virtues of cod liver oil are too well known to detail here and the combination of the iodides and oil in No. 1, and guaiacol and creosote carbonate with the iodides and oil in No. 2, present to the profession a preparation of great merit.

---

### THE NEW TREATMENT FOR GONORRHEAL INFECTIONS.

Physicians who have had any considerable experience in the treatment of gonorrhea and its complications know how stubborn many of these cases are; how, not infrequently, they resist ordinary routine methods for weeks and months. The average general practitioner encounters these cases with unpleasant forebodings. He realizes that treatment of them is more or less empirical. He experiences a sense of relief when he can bid "good-bye" to one of them—when he can discharge it as "cured."

For the reasons enumerated any new therapeutic agent which promises a fair percentage of recoveries in gonorrhea and its sequelae is certain to be accorded a warm reception by the medical profession.

Is Gonorrhea Phylacogen such an agent? There is a basis for the belief that it is. Here are some figures that seem to lend assurance: "660 cases treated; 539 recoveries; 121 failures." These figures pertain to carefully recorded cases, under observation in various sections of the United States and embracing both hospital and private practice. They include such complications as gonorrheal arthritis, chronic urethritis, vaginitis, epididymitis, orchitis, prostatitis, vesiculitis, ophthalmitis, iritis, endometritis and salpingitis. These cases were reported to Messers. Parke, Davis & Co., producers of the Schafer Phylacogens. The results point clearly to this conclusion: Gonorrhea Phylacogen is worthy of careful, serious consideration.

### TO PROMOTE DIGESTIVE TONE.

When the functional activity of the digestive tract has been impaired by concomitant diseases, and on this account, the nutritive processes are unable to serve the body effectually, Seng is always indicated. This preparation is derived from Panax (Ginseng) and is pleasant to take as well as promptly active in enticing an appetite, gently stimulating the flow of digestive secretions, and giving tone to the organs of digestion generally. Seng acts along the line of "physiological suggestion" rather than as a substitute for the digestive ferments. In other words, it promptly stimulates the secretory activity of the stomach and intestines, so that there is a speedy return to normal physiological functionating. The dose, one or two teaspoonfuls, may be administered before or during a meal. When given alone, it is preferably administered in a small amount of water. The significance of loss of appetite as a forerunner of many serious diseases, is well appreciated by the astute diagnostician and clinician. It is in such instances that Seng is valuable in greatest measure as a dependable prophylactic. By stimulating flagging digestive and assimilative functions in the beginning, it often prevents what would surely be serious and intractable nutritional disorders later.

### FUNCTIONAL DISTURBANCES OF THE LIVER.

When one stops to think of the work the liver is called upon to do in the human economy and the excessive burden so frequently placed upon it by errors of diet, faulty methods of living, etc., it is really surprising that this great organ—the largest gland in the body—does not become deranged more often than it does.

Clinical study has shown, however, that the liver is much more subject to functional disturbance than is generally realized. The opinion has been growing for some time, therefore, that not a few obscure conditions of the intestines, particularly those grouped under the term "auto-intoxication," should be attributed to sluggishness or torpidity of the liver processes.

In view of this there is an increasing demand for a cholagogue that can be relied upon to stimulate the functional activity of the liver without at the same time producing a degree of catharsis that is neither necessary or desired. Many and various are the drugs that have been tested with this in view, but practically the only one that has met the situation is that long known and widely used preparation of Chionanthus Virginica, Chionia.

This product is exceedingly effective as a stimulator of hepatic processes, and administered in proper dosage it is not only an effective cholagogue, but what is especially important, it can be relied upon to produce its effects without causing undue activity of the bowels. The prompt influence of Chionia on hepatic functions consequently gives it a broad range of usefulness, not only in overcoming liver disorders themselves, but also the various affections that are dependent on hepatic deficiency or derangement. It is indicated in acute and chronic hepatitis, catarrhal jaundice or cholangitis, biliousness, chronic intestinal catarrh, intestinal putrefaction, auto-intoxication and all maladies caused or aggravated by derangement of the liver.

The significant feature attending the use of Chionia is that it always acts by promoting or augmenting natural processes—never by superseding them. It belongs, therefore, to the class of remedies aptly designated as physiologic, for its whole action is to restore and maintain normal or physiologic conditions of the liver.

---

Now that we are able to definitely establish a diagnosis of chancre by smear and by blood test, there is no longer any reason why a genital "primary sore" should not be excised. On the contrary, the prompt removal, by this means, of its many contained spirochete is highly desirable.

